# The FARSEEING real-world fall repository: a large-scale collaborative database to collect and share sensor signals from real-world falls

**DOI:** 10.1186/s11556-016-0168-9

**Published:** 2016-10-30

**Authors:** Jochen Klenk, Lars Schwickert, Luca Palmerini, Sabato Mellone, Alan Bourke, Espen A. F. Ihlen, Ngaire Kerse, Klaus Hauer, Mirjam Pijnappels, Matthis Synofzik, Karin Srulijes, Walter Maetzler, Jorunn L. Helbostad, Wiebren Zijlstra, Kamiar Aminian, Christopher Todd, Lorenzo Chiari, Clemens Becker, for the FARSEEING Consortium

**Affiliations:** 1 Department of Clinical Gerontology, Robert Bosch Hospital, Auerbachstr. 110, Stuttgart, Germany; 2 Institute of Epidemiology and Medical Biometry, Ulm University, Ulm, Germany; 3 Department of Electrical, Electronic, and Information Engineering, University of Bologna, Bologna, Italy; 4 Department of Neuroscience, NTNU, Trondheim, Norway; 5 School of Population Health, University of Auckland, Auckland, New Zealand; 6 Department of Geriatric Research, Bethanien-Hospital/Geriatric Center at the University of Heidelberg, Heidelberg, Germany; 7 Department of Human Movement Sciences, MOVE Research Institute Amsterdam, Vrije Universiteit Amsterdam, Amsterdam, Netherlands; 8 Department of Neurodegenerative Diseases, Hertie Institute for Clinical Brain Research, University of Tübingen, Tübingen, Germany; 9 German Research Center for Neurodegenerative Diseases (DZNE), Tübingen, Germany; 10 Institute of Movement and Sport Gerontology, German Sport University Cologne, Cologne, Germany; 11 Laboratory of Movement Analysis and Measurement, Ecole Polytechnique Fédérale de Lausanne (EPFL), Lausanne, Switzerland; 12 School of Nursing, Midwifery and Social Work, University of Manchester, Manchester, UK

**Keywords:** Falls, Database, Older adults, Body-worn sensors, Accelerometer

## Abstract

**Background:**

Real-world fall events objectively measured by body-worn sensors can improve the understanding of fall events in older people. However, these events are rare and hence challenging to capture. Therefore, the FARSEEING (FAll Repository for the design of Smart and sElf-adaptive Environments prolonging Independent livinG) consortium and associated partners started to build up a meta-database of real-world falls.

**Results:**

Between January 2012 and December 2015 more than 300 real-world fall events have been recorded. This is currently the largest collection of real-world fall data recorded with inertial sensors. A signal processing and fall verification procedure has been developed and applied to the data. Since the end of 2015, 208 verified real-world fall events are available for analyses. The fall events have been recorded within several studies, with different methods, and in different populations. All sensor signals include at least accelerometer measurements and 58 % additionally include gyroscope and magnetometer measurements. The collection of data is ongoing and open to further partners contributing with fall signals. The FARSEEING consortium also aims to share the collected real-world falls data with other researchers on request.

**Conclusions:**

The FARSEEING meta-database will help to improve the understanding of falls and enable new approaches in fall risk assessment, fall prevention, and fall detection in both aging and disease.

**Electronic supplementary material:**

The online version of this article (doi:10.1186/s11556-016-0168-9) contains supplementary material, which is available to authorized users.

## Background

Falls in older people remain a major public health challenge [1]. Most of the current knowledge on risk factors is derived from epidemiological studies [2], interviews [3] and intervention studies [4]. Less than 20 % of all falls are observed by others [5, 6] and reports by fallers are often biased due to recall problems [3, 7]. To date, the contribution of objective measurements using inertial sensor-based technology to improve the understanding of the underlying mechanisms and kinematics of fall events is modest due to a lack of available real-world data.

The fall incidence rate in older populations appears to be high, varying from 0.3 falls per person-year in community-dwelling older persons to more than 3 falls per person-year in high-risk patients [8]. However, it is still very challenging to record real-world fall data with sensors. This is due to a relatively low incidence of events and limited measurement periods, as a result of battery lifetime restrictions, limited personnel resources or data storage. As an example, to capture 100 real-world falls, it would be necessary to record approximately 100,000 days of physical activity (300 person-years). If the battery lifetime is limited to 10 days, 10,000 measurement cycles would be needed. Additionally, compliance issues may arise with long measurement periods. As a consequence, most studies have failed to gather a reasonable number of objectively measured real-world fall events for older people to date.

Recently, a Canadian research group successfully recorded several hundred fall events from nursing home patients on video [9]. This study is a milestone. Although the recordings were restricted to falls in public areas, they demonstrated that video footage fills some of the knowledge gaps pertaining to the contextual factors of falls. In addition to video footage, body-worn sensors including accelerometers, gyroscopes, magnetometers, or barometers could be used to more precisely measure biomechanical parameters from real-world falls without any restriction to the fall location. A recent systematic literature review on body-worn sensors and fall detection underlined the shortage of such real-world fall signals [10]. Not more than six studies reported real-world falls, from which only one study by FARSEEING members collected a reasonable number of 20 fall events during a previous European Commission project (SENSACTION-AAL, FP6, IST Contract no. 045622).

The FARSEEING consortium and associated partners argued that a sufficient dataset of real-world falls measured by body-worn sensors could only be achieved by a joint collaborative effort of many research groups. Due to the sample size considerations as well as generalizability, this requires a willingness to share real-world fall data. Therefore, the FARSEEING project started to build a meta-database of real-world fall signals in 2012. The architecture of the database facilitates the structured collection, processing, and analysis of data related to falls and physical activity monitored by body-worn sensors, and the linking of these measures to clinical data on physical and cognitive function, medications, medical diagnoses as well as information from fall reports.

The aim of this paper is to present the structure and content of this meta-database of real-world falls and to describe the developed procedures to record, process, verify and store as well as to access this data. The results represent the status as at December 31st 2015. The collection and processing of data is ongoing and also open to further partners contributing with fall signals. The continuously updated status of the database as well as a more detailed description of the available data can be accessed via the FARSEEING website (www.farseeingresearch.eu).

## Methods

### Study population

Between the start of the project in January 2012 and December 2015 six institutions contributed with physical activity data from more than 2,000 participants from different studies and settings to the database (Table 1). This includes community-dwelling older adults as well as patient groups with high-risk of falling such as geriatric rehabilitation, nursing home, assisted living, idiopathic Parkinson’s Disease, Progressive Supranuclear Palsy (PSP), dementia, and degenerative ataxia.

**Table 1:** Recording sites contributing data from several settings to the FARSEEING meta-database

Recording site	Settings and disease groups	Status	Subjects measured^a^
Robert-Bosch Hospital (RBMF), Germany	Geriatric Rehabilitation	Ongoing	1654
	Community-dwelling	Finished	249
University of Tübingen, Germany	Ataxia	On going	16
	Idiopathic Parkinson’s Disease	On going	5
	Progressive Supranuclear Palsy (PSP)	On going	12
	Visual impairment	Planned	-
University of Nürnberg/Erlangen, Germany	Assisted living (intellectual disability)	Finished	67
German Sport University Cologne, Germany	Dementia	On going	>70
Bethanien-Hospital/Geriatric Center at the University of Heidelberg, Germany	Dementia	On going	>10
University of Auckland, New Zealand	Nursing home	Paused	19

^a^Until 31.12.2015

### Data collection

The systematic literature review on body-worn sensors and fall detection showed very heterogeneous approaches to record and store falls data [10]. Standardization was necessary to improve data quality from studies recording fall signals, but also to build up a harmonized meta-database of real-world falls. Therefore, a consensus process was established at the beginning of the FARSEEING project to agree on a standard fall data format including: (1) the sensor configuration and the fall signal description, (2) a minimum clinical dataset to describe the faller, and (3) the fall reporting [11].

1. Sensor configuration and the fall signal description: Most of the data were not primarily collected to record fall signals for the FARSEEING database, but to monitor physical activity during clinical routine or within research studies. Therefore, several different types of sensors from different manufacturers have been used. In general, the database is open to all type of body-worn sensor devices including at least accelerometer signals. The most important parameters have been included in the database to describe the technical specifications of each sensor recording such as sensor type (accelerometer, gyroscope, magnetometer), device type, sample frequency and sensor range (see Additional file 1: Table S1).
2. Minimum clinical dataset: Almost every study uses an individual predefined set of clinical variables that is not modifiable for numerous reasons. Therefore, a minimum (core) dataset of participant characteristics was used including sex, age and a functional description based upon the International Classification of Functioning (ICF) multilingual coding system (http://apps.who.int/classifications/icfbrowser/) (see Additional file 1: Table S2).
3. Fall reports: The FARSEEING consortium agreed on the following fall definition based on the ProFaNE recommendation [12]: A fall is an unexpected event in which the person comes to rest on the ground, floor, or lower level. In case of a reported or measured fall, a standardised fall report (based on an interview or oral confirmation) describing the fall event should be completed within one day after the fall event. Besides the date and time of the fall, several variables describing the fall event and the environment have been included in the fall reports (see Additional file 1: Table S3).

Furthermore, sensor signals from activities of daily living (ADL) over 24 h without a fall event have been stored for each faller, if available.

### Ethical approval & data protection

Each institution contributing with study data to the FARSEEING meta-database has to apply for an ethical approval covering the respective study. The process of data-collection combining different sources in the FARSEEING meta-database was additionally examined and approved by the Ethical Committee of the University of Tübingen (495/2012BO2) and the data protection office of the federal state of Baden-Württemberg, Germany (T 1500/231). All information entered to the meta-database is completely anonymized. Data from the same subject is labelled by a random number which cannot be linked with personalized information. Information has also been stored on an aggregated level (e.g. age truncated to full years) to avoid identification of subjects by combining several variables.

### Signal processing

During the FARSEEING project a standard operation procedure for processing the signal data has been established. The main aim is to identify date and time of each fall event within the fall signal based on the fall reports and to store all information in a uniform data format. Due to reporting imprecision, cognitive impairment of the faller, or recall problems, the reported and the true time point of the fall event might differ considerably [3, 6, 7]. Therefore, two raters independently have been screening the fall signal and try to identify the fall pattern.

Table 2 shows the standard operation procedure for the signal processing. Steps 1, 2, 5 and 6 are done by technicians with experience in signal recording and data management. The ‘fall signal identification’ and the ‘double check’ (steps 3 and 4) are performed in a blinded fashion by two independent fall signal identification experts with long standing experience in fall signal analysis.

**Table 2:** Standard operation procedure for processing the fall signals

Step	Description
1.	Data check and cleaning: The raw sensor signal, the clinical data and the fall report are checked for missing values and correct coding of the variables.
2.	Signal import: A custom-made software tool is used to import and convert the raw signals from manufacturer-specific formats to the standard FARSEEING data format (see Table 3). The sensor orientation is transferred to the uniform orientation (see Fig. 1).
3.	Fall signal identification: Based on date, time, and description of the fall event, reported by the participant during the fall interview, the fall signal is screened by the first rater. The fall event is determined by the impact if available. The beginning of the impact phase is determined as the local minimum of the acceleration signal in the vertical-axis followed by a rapid increase of the acceleration value at the impact [13]. In some cases, no impact is present, but a change in posture. In this case the fall event is defined by the first change in posture. If it is possible to identify the fall event, the status is set to ‘verified fall.’
4.	Double check: Step 3 is performed by the second rater in a blinded fashion. In case of disagreement the signal is discussed in an expert panel (including experts of the FARSEEING consortium). If the experts or the expert panel agree on the fall event, the status is set to ‘finally verified.’ If there is no agreement on the fall event in the expert panel, the status is set to ‘non-verifiable fall.’
5.	Fall signal extraction: The fall signal is stored in a separate file according to the FARSEEING standard fall signal format described below. The pre-fall time is set to 10 min and the post-fall time to at least 10 min or until a recovery movement was observed.
6.	Data up-load: The extracted signal, the clinical data and the fall report are entered in the FARSEEING meta-database. To completely anonymize the data, variables are transformed to an aggregated level and any identification code is removed.

### Signal file format

**Fig. 1:**
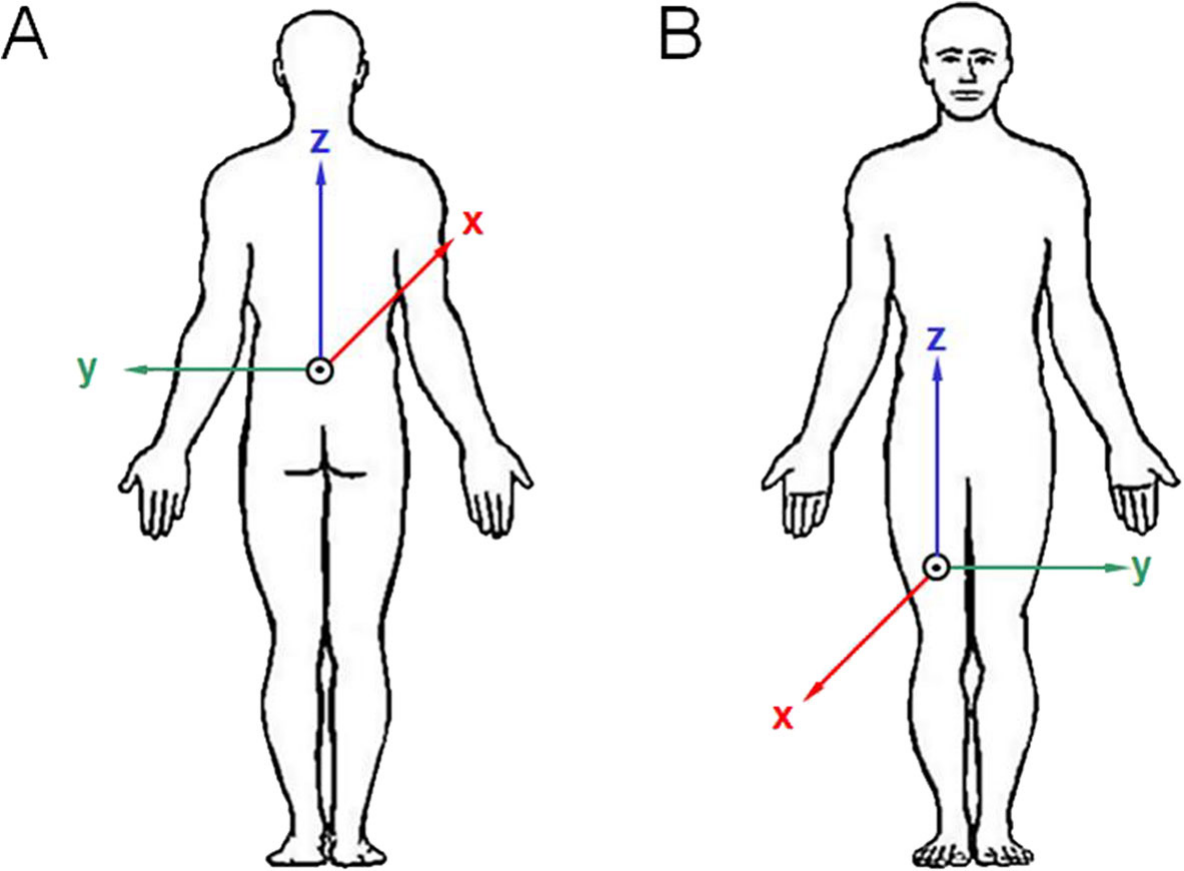
Uniform fall signal orientation for L5 (**a**) and thigh location (**b**)

All signals have been imported and converted to a uniform MATLAB mat-file. The fall signal file name includes an anonymized identifier and the fall date with the following structure: Random ID-Fall number-Year-Month-Date-Hour-Minute-Seconds.mat. Table 3 presents the structure of each fall signal file. It includes the relative time since the start of the measurement, the absolute time in MATLAB time format, the values of the sensor recordings and a fall indicator variable with a verification certainty score (definition see below). The MATLAB time format uses an absolute time scale based on a reference date, counted in days. The reference date is January 1st 0000. Time is represented as a relative number based on one day (e.g. 12 am ≡ 0.5). Additionally, the sensor orientation of all signals has been converted to a uniform orientation based on the reported sensor placement (Fig. 1). The sensor orientation was defined based on the reported sensor placement. None of the contributing studies performed a standardized calibration of the sensor orientation at the beginning of the measurements.

**Table 3:** Signal file format

Column	Description
1	Relative time in seconds
2	Absolute time in MATLAB time format
3	Acceleration signal along the x-axis [m/s^2^]
4	Acceleration signal along the y-axis [m/s^2^]
5	Acceleration signal along the z-axis [m/s^2^]
6	Gyroscope signal along the x-axis [°/s]
7	Gyroscope signal along the y-axis [°/s]
8	Gyroscope signal along the z-axis [°/s]
9	Magnetometer signal along the x-axis [μT]
10	Magnetometer signal along the y-axis [μT]
11	Magnetometer signal along the z-axis [μT]
12	Fall indicator value

### Fall indicator and verification certainty score

A fall indicator variable was created to mark the start of the impact phase of the fall events in the signal file. As mentioned before, the reported date and time of the fall event and the date and time of the fall event identified by the raters in the sensor signal could differ significantly. Therefore, a number between 1 and 4 indicates the start of the impact phase. Furthermore, the value of the number shows the degree of verification certainty based on the lag between the reported date and time and the identified date and time in the sensor signal as well as the correspondence of the reported pre-fall activity (e.g. walking) and orientation with the signal data. Table 4 shows the definition of the verification certainty categories. The value of all other time samples in the signal file was set to 0.

**Table 4:** Categories of verification certainty

Verification certainty	Description of the categorisation based on the correspondence of timing between reported and identified date and time as well as on the correspondence between description of the fall event and the signal data
Not verifiable	Fall date of the sensor signal does NOT correspond with the reported date OR more than one possible fall signals have been identified at the same date.
1	Fall date of the sensor signal corresponds with the reported date AND the description of pre-fall activity and orientation does NOT correspond with the sensor signals.
2	Fall date of the sensor signal corresponds with the reported date AND the description of pre-fall activity and orientation corresponds with the sensor signals OR Time lag between reported and identified date and time is ±60 min AND the description of pre-fall activity and orientation does NOT correspond with the sensor signals.
3	Fall date and time of the sensor signal corresponds with the reported time of the day such as morning, noon, afternoon, evening, or night AND the description of pre-fall activity and orientation corresponds with the sensor signals.
4	Time lag between reported and identified date and time is ±60 min AND the description of pre-fall activity and orientation corresponds with the sensor signals.

A printable documentation of all finally processed falls is available and includes a summary of the faller characteristics and the fall report as well as a description of the sensor configuration and printed signals of all sensors (accelerometer, gyroscope and magnetometer) (for an example see Additional file 2).

## Results

**Fig. 2:**
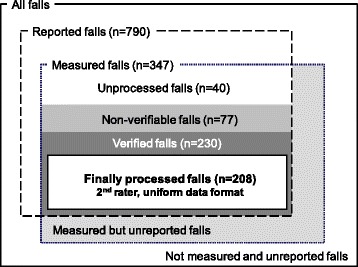
Stages of the signal processing and verification process

Figure 2 shows the different stages and corresponding numbers of the verification process. Until the 31st December 2015 a large amount of falls has been reported to the FARSEEING consortium. From these reported falls, 347 falls have been recorded by sensors. From these recorded falls 208 falls have been independently verified by two independent raters. About 5 % of the considered events have been discussed by the expert panel, because the two raters did not agree. The distribution of the verification certainty from 1 to 4 for the identified fall events was 5.8 %, 19.7 %, 3.8 %, and 70.7 %, respectively. Currently, it was not possible to find and verify reported fall events in 77 signal files, even after the expert panel consultation. As the collection and processing of data is still ongoing the continuously updated status of the database as well as a more detailed description of the available data can be accessed via the FARSEEING website (www.farseeingresearch.eu).

Currently, the verified dataset includes 94 fallers with a mean age of 76.1 (SD = 12.6) years (54.3 % women). About 73 % of the signals were sampled at 100 Hz and 27 % at 20 Hz (Table 5). Signals with accelerometers, gyroscopes and magnetometers were available for 121 (58 %) of the measured fall events. The most common sensor placement was the lower back position at L5 (72 %), while all other signals were recorded at the thigh.

**Table 5:** Technical characteristics of the fall data (n = 208)

Description		n (%)
Sample rate	20 Hz	56 (27 %)
	100 Hz	152 (73 %)
Sensor configuration	Acc	72 (35 %)
	Acc, gyro	15 (7 %)
	Acc, gyro, mag	121 (58 %)
Sensor location	L5	150 (72 %)
	Thigh	58 (28 %)

**Fig. 3:**
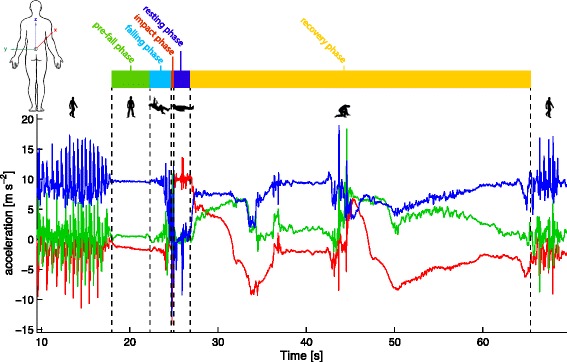
Fall signal example with labelled activities and fall phases

Figure 3 shows a real-world fall signal example (acceleration) with labelled activities and fall phases. The sensor (Samsung Galaxy S3) was attached at the lower back by means of an elastic belt, sampling at 100 Hz. The faller reported a backwards fall while pushing the door opener. The person was upright at the beginning, indicated by the vertical axis (blue) showing 10 m/s^2^, including some walking. During the fall the vertical signal changes to 0 m/s^2^ and the anterior-posterior axis (red) to 10 m/s^2^, indicating a backward fall. After a short period of resting, the person recovered with an intermediate resting position and continued walking.

## Discussion

The FARSEEING falls database is the first systematic multi-centre research database collecting real-world fall sensor signals measured with body-worn devices. The database currently provides more than 200 verified real-world fall events for analyses. This is at present the largest collection of real-world falls using inertial sensors. The database allows new and innovative analyses of various research questions and the application of more complex analytical methods such as machine learning and pattern recognition to better understand the biomechanics of falls.

Based on this foundation, the collection and processing of falls data is still ongoing and will be continued to further enrich the database. Research groups that already collected fall signals with body-worn sensors or plan studies to collect signals from real-world falls are invited to join the consortium. The FARSEEING consortium also aims to include fall signals from further types of sensors, such as barometers and heart rate monitors.

We see this data collection as a big step forward in the possibility to analysis and understand falls in older persons. However, some methodological issues have to be considered by database users. First, the fall events have mostly been collected in groups with high risk of falling and within selected populations. Falls from community-dwelling older persons are currently under-represented in the database when compared to the overall number of falls in the general populations. However, considering specific and well-defined disease groups might show more distinct patterns and help to better understand specific aspects of fall events, e.g. the impact of coordination disturbances on falls can be optimally studied in the paradigmatic group of subjects with ataxia.

Second, reported and recorded falls overlap, but do not completely represent the same fall events. Some fall events were reported but not recorded. Possible reasons are that the sensor was started after or stopped before the fall due to organisational issues or due to battery life-time. Technical limitations, such as a low sampling rate, might have prevented the detection of an impact and the verification of a reported fall. However, in most cases a fall event was also indicated by a sudden change in posture. More problematic are fall events which were recorded, but not reported. Data that are categorised as ‘non-fall data’ or ‘ADL data’ might actually include fall events. It is not possible to estimate the amount of underreporting and mark those fall events in the collected data, yet. Even if applying a very tight fall assessment, underreporting might occur, especially in populations with cognitive impairment [6, 12]. It should be noted that when assessing the specificity of fall detection algorithms, a false positive event might actually be a true positive event and therefore bias the results.

Third, due to the different data sources and varying assessment methods clinical data was not available for all contributing cohorts. To reduce this problem in future studies, it is recommended to consider the proposed data recording structure [11] when developing new studies, even if fall recording is not the primary task.

The FARSEEING consortium aims to share the falls data with other researchers. A dataset of 20 selected fall events is available on request via the project website (www.farseeingresearch.eu). Researchers are also invited to collaborate with the FARSEEING consortium on specific research questions and get access to the full FARSEEING meta-database. A scientific board decides about each proposal for collaboration. The consortium does not share data with projects having primarily commercial interests. However, collaborative projects between industry and the FARSEEING research group, e.g. for algorithm development and validation, are possible. More information about the data sharing policy can be found on the FARSEEING website (www.farseeingresearch.eu).

## Conclusion

In conclusion, the FARSEEING meta-database is currently the largest collection of real-world falls using inertial sensors. It will help to substantially improve the understanding of falls and enable new approaches in fall risk assessment, fall prevention, and fall detection.

## Additional files

Additional file 1: Table S1. Technical specifications. **Table S2**. Minimum clinical dataset. **Table S3**. Fall reporting. (DOCX 28 kb)
Additional file 2: Fall report. (PDF 622 kb)

## Abbreviations

ADL: Activities of daily living
FARSEEING: Fall repository for the design of smart and self-adaptive, environments prolonging independent living
ICF: International classification of functioning
L5: The fifth lumbar vertebra
PSP: Progressive supranuclear palsy
SD: Standard deviation
