# Supplementary material for: The FARSEEING real-world fall repository: a large-scale collaborative database to collect and share sensor signals from real-world falls
Source: Eur Rev Aging Phys Act. 2016 Oct 30;13:8. doi: 10.1186/s11556-016-0168-9 (PMC5086409; doi:10.1186/s11556-016-0168-9)
Supplement: Additional file 1: Table S1. — Technical specifications. Table S2. Minimum clinical dataset. Table S3. Fall reporting. (DOCX 28 kb) [file 11556_2016_168_MOESM1_ESM.docx]

Additional file 1

Table S1. Technical specifications

| Variable |  | Description |
| --- | --- | --- |
| Signal ID |  | Unique signal number or description (e.g. ‘x-axis’ or ‘signal 1’) |
| Sensor ID |  | Unique number of the sensor which generated the signal |
| Device ID |  | Unique device identification number or name |
| Date & time |  | Date and time of start of recording [DD.MM.YYYY:HH:MM:SS] |
| Device manufacturer |  | The manufacturer of the sensor device (e.g. SAMSUNG) |
| Model |  | The unique model identification (e.g. Hybrid) |
| Firmware |  | The unique firmware identification (e.g. V1.02) |
| Sensor manufacturer |  | The manufacturer of the sensor unit (e.g. BOSCH) |
| Sensor model |  | The unique model identification (e.g. BMA150) |
| Sensor type |  | The type of sensor (e.g. gyroscope) |
| Sample rate |  | Sample rate of the sensor signals |
| Unit |  | Unit on which the sensor is calibrated |
| Resolution |  | Resolution of the sensor signal |
| Lower limit |  | Minimal detectable value |
| Upper limit |  | Maximal detectable value |
| Sensor placement |  | On which body segment was the sensor placed (see ‘sensor placement and attachment’) |
| Sensor attachment |  | Method how the devices/sensor was attached to the body of the participant (e.g. body fixed, body worn, external)  Since the different sensors of a device could be separate the type of attachment will be assessed per sensor. |
| Signal orientation |  | Orientation of the sensor signal relative to the global reference system (see ‘sensor placement and attachment’) |

Table S2. Minimum clinical dataset

| Variable |  | Description |
| --- | --- | --- |
| Sex |  | Male or female |
| Age |  | 0-99 and > 99 |
| Body weight |  | Kg |
| Body height |  | Cm |
| Fall history |  | Yes or no  If yes, number of fall during the last year |
| Assistive device |  | Yes or no |
| Motor impairment -  standing capacity |  | ICF coding d 4104 |
| Motor impairment -  sit-to-stand transfer |  | ICF coding b 730 (change of body position) |
| Motor impairment -  gait |  | ICF coding b 770 |
| Cognitive impairment |  | ICF coding b 164 |
| Emotional disturbance (e.g. depression and  fear of falling) |  | ICF coding b 152 |
| Visual impairment |  | ICF coding b 210 |

Table S3. Fall reporting

| Variable |  | Description |
| --- | --- | --- |
| Date & time |  | Date & time of the fall – approximate hour |
| Fall description |  | Free text describing the fall event (patient and/or proxy report) |
| Witnessed or unwitnessed |  | Did another person observe the fall? – Yes or no |
| Pre-fall activity |  | Activity before the fall using movement categories (e.g. sitting, lying, standing, walking, turning) |
| Environmental information |  | Environmental information – categorical (e.g. floor surface, obstacles) |
| Use of assistive device |  | Did the faller use an assistive device directly before the fall? – Yes or no  If yes, categorical description, i.e. rollator, stick, wheelchair etc. |
| Loss of consciousness, epilepsy, stroke hypoglycemia |  | Yes or no |
| Initial falling direction |  | Categorical: forward, backward, side (left or right), straight down |
| Multiple impact e.g. hit wall or furniture “broken fall impact” |  | Yes or no |
| Location of the fall event |  | Outdoor (e.g. public transport, traffic accident) or indoor (e.g. bedroom, toilet, dining room, staircase, other) |
| Duration of lying or resting |  | In minutes (approximate) |
| Got up without help |  | Yes or no |
| Contact to medical or care institutions |  | Yes or no |
| Injury classification |  | Type  A Fracture, head or internal injury requiring accident and emergency or inpatient treatment  B Wounds, bruises, sprains, cuts requiring a medical/health professional examination such as physical examination,  x-ray, suture  Reduction in physical function (e.g. due to pain, fear of falling) for at least 3 days.  C no consequences |
| Injury location |  | Which part of the body segment was affected. |
