# Supplementary material for: The FARSEEING real-world fall repository: a large-scale collaborative database to collect and share sensor signals from real-world falls
Source: Eur Rev Aging Phys Act. 2016 Oct 30;13:8. doi: 10.1186/s11556-016-0168-9 (PMC5086409; doi:10.1186/s11556-016-0168-9)

Fall example

Setting: Ataxia

Signal file: F_93807530-06-2014-02-19-09-05-49.mat

Personal characteristics

Gender: female

Age: 57 yrs

Height: 167 cm

Weight: 70 kg

Sensor characteristics

Device: Samsung Galaxy S3

Location: L5

Sensor type(s): acc, gyro, mag

Unit(s): m/s², °/s, μT

Sample rate: 100 Hz

Fall report

Fall time reported: 19.02.2014 09:15:00

Fall time signal: 19.02.2014 09:05:49

When washing the hair, towel slipped over the forehead and covered the eyes. Due to loss of vision, subject fell backwards.

Witnessed: No

Assistive device: No

Reported pre-fall activity: standing/grasping

Reported fall direction: backwards

Indoor/outdoor: Indoor

Place of fall: -

Multiple impact: unknown

Fall on: bathtub

Got up without help: Yes

Injury: Yes

Injury classification: B

Injury location: head

Injury description: laceration at the back of the head

Adopted measures: no medical contact

Fall example
Time window: 60 s

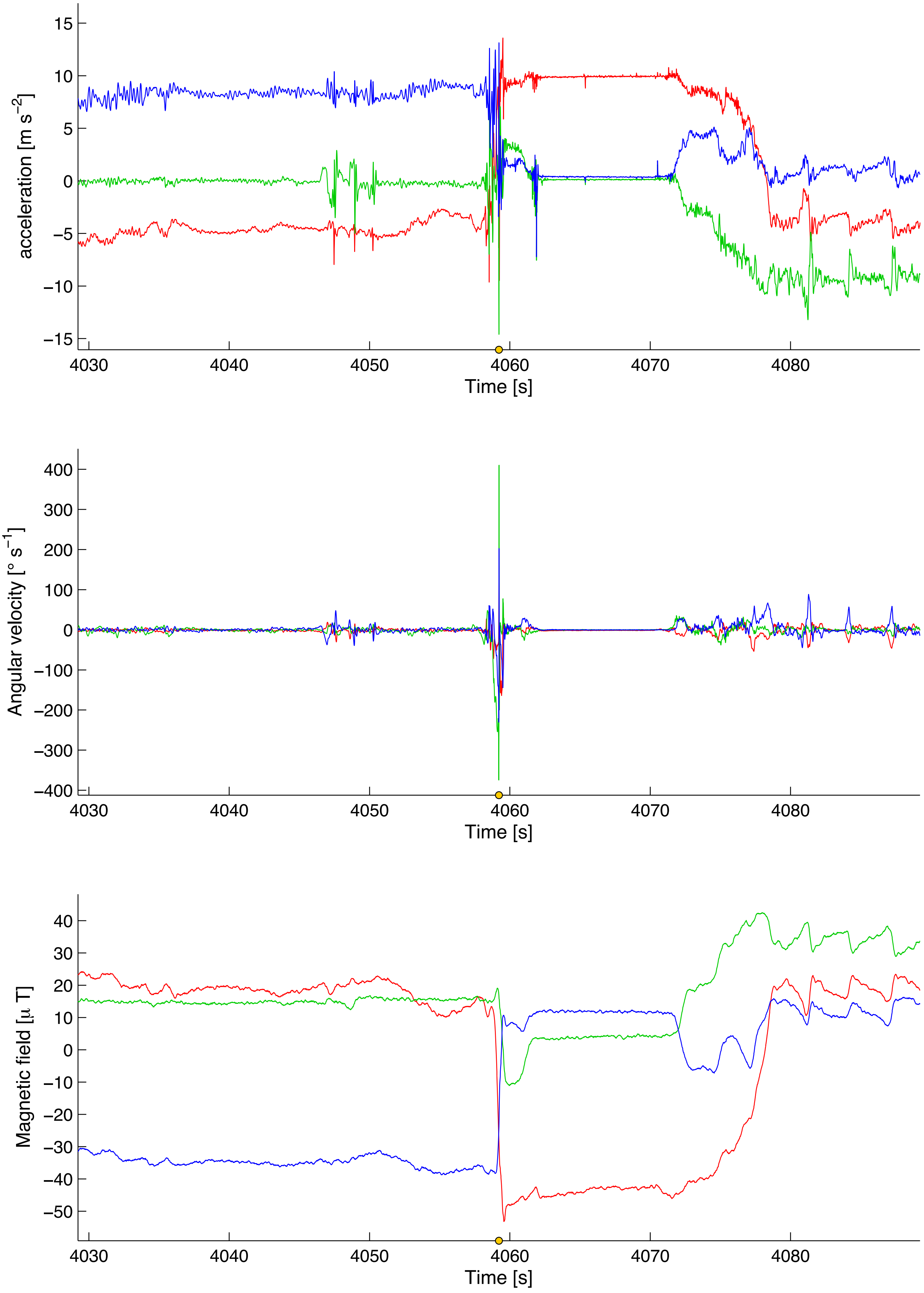

Supplement: Additional file 2: — Fall report. (PDF 622 kb) [file 11556_2016_168_MOESM2_ESM.pdf]
